# Draft Genome Sequence of Lactiplantibacillus pentosus AWA1501, Isolated from Awa-bancha

**DOI:** 10.1128/MRA.00518-21

**Published:** 2021-07-29

**Authors:** F. Ito, R. Niwa, Y. Syaputri, Y. Ikagawa, T. Mizuno, M. Horie, H. Iwahashi

**Affiliations:** a Faculty of Applied Biological Sciences, Gifu University, Gifu, Japan; b The United Graduate School of Agricultural Science, Gifu University, Gifu, Japan; c Health Research Institute, National Institute of Advanced Industrial Science and Technology (AIST), Takamatsu, Japan; Indiana University, Bloomington

## Abstract

Lactiplantibacillus pentosus AWA1501 was isolated from the traditional Japanese tea Awa-bancha. Previous studies have reported that this species becomes predominant after the anaerobic fermentation process. In this study, we report the whole-genome sequence of this strain. The draft genome sequence comprises 3,714,221 nucleotides and 3,374 coding DNA sequences, with an average G+C content of 46.02%.

## ANNOUNCEMENT

Tea is one of the most common beverages consumed worldwide. There are different varieties of tea, such as unfermented, semifermented, fermented, and postfermented, depending on the fermentation processes involved. Fermented tea is produced with the oxidative enzymes in the tea leaves, whereas postfermented tea requires microbial fermentation. Awa-bancha is a type of postfermented tea manufactured in Nara and Kamikatsu, Tokushima Prefecture, Japan. Anaerobic fermentation, mainly using lactic acid bacteria, is crucial for the production of Awa-bancha. Previous research has shown that two species of bacteria, Lactiplantibacillus plantarum and Lactiplantibacillus pentosus, are the predominant species which play an essential role in the fermentation of Awa-bancha (1, 2). These bacteria generate lactic acid, which gives a unique acidity and flavor to Awa-bancha compared to that of green tea. It also improves the durability of the tea. In this study, we sequenced the genomic DNA of *L. pentosus* isolated from Awa-bancha.

*L. pentosus* AWA1501 was isolated from fermented Awa-bancha tea leaves in Kamikatsu, Tokushima Prefecture. Wet tea leaves (0.5 g) were added to sterile distilled water at a concentration of 0.1 mg/ml. The suspension was subsequently diluted with sterile distilled water and spread onto MRS agar plates. These plates were incubated at 30°C for 1 to 2 days under anaerobic conditions using the AnaeroPack system (Mitsubishi Gas Chemical Co., Inc., Tokyo, Japan). Single colonies were picked and inoculated into 10 ml of MRS broth in a test tube with a screw cap. Each isolate was incubated at 30°C for 1 to 2 days. The genus of the isolated bacteria was identified by sequencing 16S rRNA and performing a BLAST search of the NCBI nucleotide database. Multiplex PCR of the *recA* gene was used to determine the species (1). The stock cultures were stored at −80°C in MRS broth containing 30% glycerol. Genomic DNA extraction was done using the AWA1501 stock culture. Stock cultures were picked and inoculated into MRS broth and incubated for 16 h. Genomic DNA extraction was carried out using the Extrap soil DNA Plus kit v2 (Nippon Steel and Sumikin EcoTech Corporation). The DNA sample was submitted to the Gifu University Next Generation Sequencer (NGS) service, which employs Illumina MiSeq sequencing. The libraries were prepared for the Illumina MiSeq instrument (300-bp paired-end [PE] format) using the Nextera XT DNA library prep kit.

From this library, 1,344,167 reads were obtained. An initial quality check was performed using FastQC v0.11.9 (http://www.bioinformatics.babraham.ac.uk/projects/fastqc/). The adaptors and low-quality reads were trimmed using Fastp v0.20.1 with a threshold of Q25 (3). After trimming, the reads were assembled *de novo* using Unicycler v0.4.8 and evaluated using QUAST v5.0.5 (4, 5). A quality check of the assembly was conducted using Benchmarking Universal Single-Copy Orthologs (BUSCO) v5.1.2 with the *Lactobacillales* data set (6). Gene annotation was conducted using DFAST v1.2.4 in the Web application version with the database (DB) type set to lactic acid bacteria (7). Default parameters were used for all software unless otherwise specified.

The resulting assembly had 91 contigs with 3,714,221 bp, a mean G+C content of 46.02%, an *N*_50_ value of 193,547 bp, and a total of 3,374 genes. The BUSCO analyses showed 400 complete single-copy genes and 1 completely duplicated gene. The draft genome was visualized using Bandage v.0.8.1 (8), and two independent circular sequences with approximately 3,800 bp were found.

The genome sequence presented in this work is a valuable resource for understanding why *L. pentosus* becomes a predominant species during the production process of Awa-bancha.

### Data availability.

The BioSample, DRA/SRA, and BioProject accession numbers for the sequence reported here are SAMD00284712, DRA011678, and PRJDB11289, respectively. The GenBank accession number of the deposited genome is BOUG01000001.1.
