# Correction for Ito et al., “Draft Genome Sequence of Lactiplantibacillus pentosus AWA1501, Isolated from Awa-bancha”

**DOI:** 10.1128/mra.00064-23

**Published:** 2023-02-27

**Authors:** Fumiya Ito, Ryo Niwa, Yolani Syaputri, Yuichiro Ikagawa, Tomofumi Mizuno, Masanori Horie, Hitoshi Iwahashi

## AUTHOR'S CORRECTION

Volume 10, no. 30, e00518-21, 2021, https://doi.org/10.1128/MRA.00518-21. The authors’ names were spelled incorrectly in the byline in the original article. The byline should appear as shown in this Author Correction.

